# The effect of self-identified arm dominance on exercising forearm hemodynamics and skeletal muscle desaturation

**DOI:** 10.1371/journal.pone.0305539

**Published:** 2024-06-17

**Authors:** Jacob L. Schwartz, Trishawna A. Fongwoo, Robert F. Bentley

**Affiliations:** Faculty of Kinesiology & Physical Education, University of Toronto, Toronto, Ontario, Canada; Niigata University of Health and Welfare, JAPAN

## Abstract

The human forearm model is commonly employed in physiological investigations exploring local vascular function and oxygen delivery; however, the effect of arm dominance on exercising forearm hemodynamics and skeletal muscle oxygen saturation (SmO_2_) in untrained individuals is poorly understood. Therefore, the purpose of this study was to explore the effect of self-identified arm dominance on forearm hemodynamics and SmO_2_ in untrained individuals during submaximal, non-ischemic forearm exercise. Twenty healthy individuals (23±4 years, 50% female; 80% right-handed) completed three-minute bouts of supine rhythmic (1 second contraction: 2 second relaxation duty cycle) forearm handgrip exercise at both absolute (10kg; 98N) and relative (30% of maximal voluntary contraction) intensities in each forearm. Beat-by-beat measures of forearm blood flow (FBF; ml/min), mean arterial blood pressure (MAP; mmHg) and *flexor digitorum superficialis* SmO_2_ (%) were obtained throughout and averaged during the final 30 seconds of rest, exercise, and recovery while forearm vascular conductance was calculated (FVC; ml/min/100mmHg). Data are Δ from rest (mean±SD). Absolute force production did not differ between non-dominant and dominant arms (97±11 vs. 98±13 N, p = 0.606) whereas relative force production in females did (69±24 vs. 82±25 N, p = 0.001). At both exercise intensities, FBF_RELAX_, FVC_RELAX_, MAP_RELAX_, and the time constant tau for FBF and SmO_2_ were unaffected by arm dominance (all p>0.05). While arm dominance did not influence SmO_2_ during absolute intensity exercise (p = 0.506), the non-dominant arm in females experienced an attenuated reduction in SmO_2_ during relative intensity exercise (-14±10 vs. -19±8%, p = 0.026)–though exercise intensity was also reduced (p = 0.001). The present investigation has demonstrated that arm dominance in untrained individuals does not impact forearm hemodynamics or SmO_2_ during handgrip exercise.

## Introduction

The completion of physical activity requires the cardiovascular system to match the delivery of oxygen to the muscle’s oxygen demand. Oxygen delivery represents a combination of convective (i.e., muscle blood flow) and diffusive (i.e., red blood cell desaturation) components in which blood is delivered to capillaries at the active skeletal muscle and oxygen subsequently diffuses into the active skeletal muscle [1–3]. During small muscle mass exercise, such as one-legged knee extension or forearm handgrip, the pumping capacity of the heart is not approached, and therefore, local control of blood flow determines the rate of convective oxygen delivery to active skeletal muscle [4]. As such, the human forearm model is commonly employed in physiological investigations exploring vascular function and oxygen delivery [5–8].

Studies utilizing forearm handgrip exercise often assess the left [9,10] or right [6,11] arm exclusively for both consistency and methodological simplicity. However, individuals tend to have a stronger dominant compared to non-dominant arm [12]; a difference often attributed to habitual training associated with completion of activities of daily living [13]. Furthermore, chronic forearm training arising from racquet sports participation results in an enlarged brachial artery diameter in the dominant arm [14,15] and increased dominant arm peak blood flow following ischemic [16] and non-ischemic [15] handgrip exercise. In untrained individuals, conflicting reports suggests that endothelial function in response to reactive hyperemia is either independent [17] or dependent [18] on arm dominance. Further, while some evidence suggests that endothelial function is correlated between limbs [17], a lack of correlation between limbs has also been observed with grip strength playing an important role [18]. Pharmacological induced dilatory capacity and resting brachial artery diameter do not appear different between arms [16] with limited evidence suggesting peak blood flow in response to ischemic handgrip exercise is also not different [16]; however, it is unknown whether arm dominance within untrained individuals modulates forearm hemodynamics during unperturbed forearm exercise, which is more physiologically relevant than experimentally induced vasodilation and ischemia. Of note, the efficacy of matching oxygen delivery to the active muscle’s oxygen demand during exercise following a hemodynamic perturbation [9,19,20] is heterogeneous with potential implications of arm dominance unclear.

While hemodynamic differences between the upper and lower limbs have been addressed [21], there is a paucity of data within limb (e.g., upper) describing potential effects of dominance and a complete absence of potential effects on skeletal muscle oxygen saturation (SmO_2_). Therefore, the purpose of this study was to explore the effect of self-identified arm dominance on forearm hemodynamics and SmO_2_ in untrained individuals during submaximal, non-ischemic forearm exercise. We hypothesized that during absolute intensity exercise, forearm blood flow (FBF), vasodilation and SmO_2_ in addition to the temporality of hemodynamics would not differ. We further hypothesized that during relative intensity exercise, FBF and vasodilation would be greater in the dominant arm arising from greater handgrip force, while SmO_2_ and the temporality of hemodynamics would not differ.

## Materials and methods

### Participants

Twenty healthy, recreationally active (<3 hours/week of structured exercise) individuals participated in this study (23±4 years, 50% female; 80% right-handed) between June 12 and July 21, 2023. Females participated throughout menstrual cycle phases (50% follicular phase, 40% luteal phase, 10% intrauterine device). The University of Toronto Health Sciences Research Ethics Board approved this study (#41525) according to the terms of the Declaration of Helsinki and all participants provided written informed consent before their participation. Health status was confirmed by the completion of the Get Active Questionnaire [22]. All participants were 18–35 years of age with a body mass index < 29.9 kg/m^2^, normotensive, non-smokers, and had no history of cardiovascular disease or specific forearm training (e.g., tennis, racquetball). Participants were excluded if they had a skinfold thickness above the *flexor digitorum superficialis* greater than 12.5 mm, had a brachial artery bifurcation in their upper arm at the ultrasound site, a clear image of their brachial artery could not be obtained or they previously suffered from COVID-19 that required hospitalization.

### Study design

This was a within participant study in which submaximal supine rhythmic forearm handgrip exercise was completed using each forearm. Participants reported to a temperature-controlled (20–21˚C) laboratory for a single data collection session. Participants were asked to refrain from exercise for 24 hours, alcohol, caffeine and/or any energy-altering substances for twelve hours, and food for four hours prior to their laboratory visit.

### Standard anthropometric data

Upon arrival at the laboratory, standard anthropometric measurements were obtained from each participant. Biological sex, handedness, age, height and weight were obtained as well as forearm girth and skinfold tissue thickness above the *flexor digitorum superficialis*. Forearm measurements were obtained from each forearm. To determine handedness, participants were prompted with questions from the Edinburgh Handedness Inventory [23] to objectively identify handedness in activities of daily living such as writing, drawing or using utensils. A seven day physical activity questionnaire adapted from Sarkin et al. [24] was completed to confirm current physical activity habits.

### Submaximal forearm exercise protocol

#### Rhythmic forearm handgrip exercise

**Fig 1:**
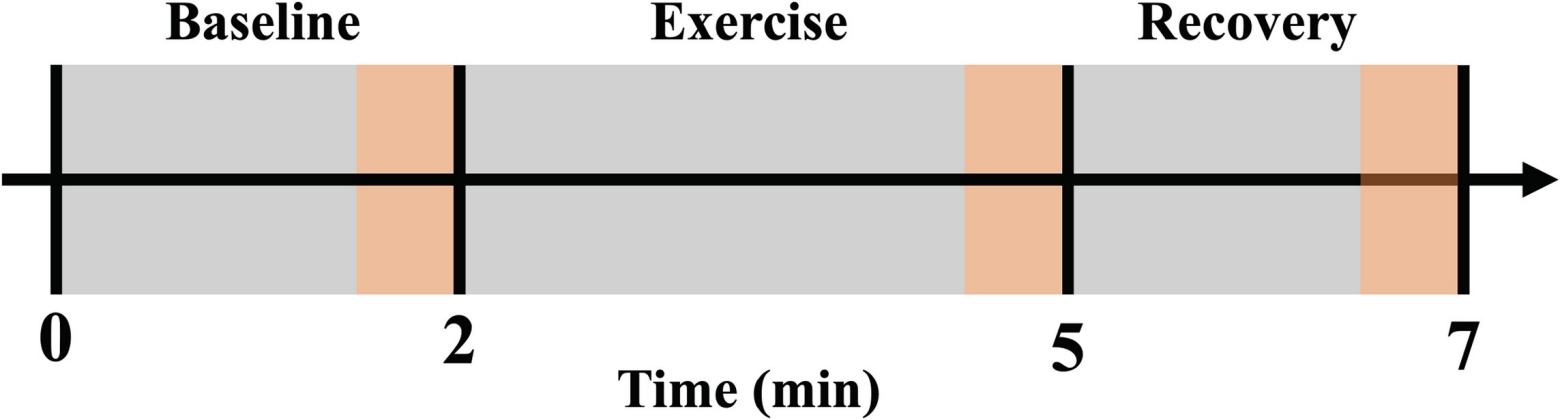
Experimental protocol. Participants completed supine, rhythmic (1 second contraction: 2 second relaxation duty cycle) forearm handgrip exercise using each forearm. Measurements were monitored over a two-minute baseline period, followed by a three-minute exercise period, followed by a two-minute recovery period. The acquisition of forearm blood flow, mean arterial pressure, forearm vascular conductance and skeletal muscle oxygen saturation occurred during the final 30 seconds of baseline, exercise, and recovery (highlighted in orange).

Participants completed three minutes of rhythmic (1 second contraction: 2 second relaxation duty cycle) forearm handgrip exercise using each forearm at both absolute and relative intensities. Participants were in a supine position with the exercising arm supinated at heart level and abducted ∼90° from the torso. The non-exercising arm was relaxed in a neutral position adducted along the side of the torso. A total of 4 exercise conditions were completed and separated by ~10 minutes or until brachial artery mean blood velocity returned to resting baseline. The order of conditions was randomized and counterbalanced. Absolute intensity exercise was performed at 10 kg (98 N) while relative intensity exercise was performed at 30% of each participant’s forearm specific maximal voluntary contraction (MVC). Exercise completion and forearm handgrip force production was guided by visual force output, metronome cues and verbal support. Two-minutes of resting baseline before and two-minutes of recovery after exercise were recorded (Fig 1).

#### Maximum voluntary contraction

Prior to the exercise protocol, three 1 second MVCs with each forearm were completed with ~1 minute of rest in between each contraction. The greatest MVC from each forearm was selected for relative intensity prescription.

### Central hemodynamics

A three-lead electrocardiogram was used to measure heart rate. A pulse oximeter (ChoiceMMed; Beijing Choice Electronic Technology Co., Ltd) was placed over the index finger of the non-exercising hand at heart level and was used to confirm stable oxygen pulse saturation (SpO_2_) during exercise. A finger photoplethysmograph (Finometer PRO; Finapres Medical Systems) on the middle finger of the non-exercising hand at heart level was used to measure mean arterial blood pressure (MAP) (Finapres Medical Systems). All variables were measured continuously.

### Forearm blood flow

A combination of echo and Doppler ultrasound was used to measure FBF. An 11L linear transducer (3–11 MHz) was positioned over the brachial artery proximal to the antecubital fossa in two-dimensional B-mode (GE Vivid E9 Imaging System, GE Medical; Horten, Norway) to image the diameter of the brachial artery of the exercising arm. The insonation angle of the probe to the artery was <60°, and the Doppler sample volume was adjusted to encapsulate the entire vessel lumen, with the vessel running parallel to the angle correction cursor. All data was recorded continuously. The continuous Doppler audio signal was converted to real-time blood velocity waveforms using a validated Doppler audio convertor [25] and sampled continuously at 200 Hz (Powerlab; ADInstruments Inc., Bella Vista, NSW, Australia) and stored (LabChart; ADInstruments Inc.) for offline analysis.

### Skeletal muscle oxygen saturation

Skeletal muscle oxygen saturation of the *flexor digitorum superficialis* was measured by continuous wave near-infrared spectroscopy (Moxy NIRS; Fortiori Design LLC, Hutchinson, MN, USA) at 2 Hz. This device employs four wavelengths of near-infrared light (680, 720, 760, and 800 nm) with a penetration depth of 12.5 mm based upon light detector and emitter distances and provides relative changes in the proportional concentrations of oxygenated and deoxygenated hemoglobin/myoglobin (SmO_2_ = oxy-[Hb/Mb]/total [Hb/Mb], %). The device was secured to the skin over the *flexor digitorum superficialis* with adhesive tape and covered with a light-shield. SmO_2_ was measured continuously.

### Data analysis

#### Brachial artery diameter and blood velocity

Brachial artery images were stored in a .mp4 format on a separate computer and analyzed using automated edge-detection software (MAUI, Hedgehog Medical Inc. Waterloo, Canada). Brachial artery blood velocity was captured during the final 30 seconds of exercise in each condition. Further, within the final 30 seconds of exercise, brachial artery blood velocity was captured within full cardiac cycles during the relaxation phase of the forearm handgrip contraction-relaxation duty cycle and averaged over the final 10 duty cycles.

#### Calculated cardiovascular variables

FBF was derived from the combined brachial artery diameter and mean blood velocity measurements. FBF was calculated as

$$(\text{mean}\;\text{blood}\;\text{velocity}\left.(\text{cm}/\text{s})\cdot \pi {\left[\text{brachial}\;\text{artery}\;\text{diameter}(\text{cm})/2\right]}^{2}\right)\cdot 60\text{s}/\text{min}$$

Forearm vascular conductance during relaxation periods (FVC_RELAX_) quantifies the pressure-flow relationship during relaxation between contractions resulting solely from vasodilation and was calculated as

$${\text{FBF}}_{RELAX}/\text{MAP}\times 100\text{mmHg}$$

as described previously [9].

#### Data acquisition periods

All beat-by-beat measures of FBF, MAP and SmO_2_ were averaged during the final 30 seconds of baseline, exercise and recovery (Fig 1). In addition, FBF during relaxation in between contraction-relaxation duty cycles was used to calculate FVC_RELAX_ during the final 10 duty cycles of each exercise condition. FBF and SmO_2_ were subsequently acquired beat-by-beat and averaged into 3 second intervals reflective of the contraction-relaxation duty cycle for time course analysis. Furthermore, SmO_2_ nadirs during exercise were identified as the lowest average 3 second time interval.

#### Time course analysis

Time course analyses were completed for FBF (n = 17; 59% female) and SmO_2_ (n = 18; 56% female). Three participants were excluded due to a beat-by-beat data extraction issue. FBF was fit with a mono-exponential model in order to assess the time required to reach 63% of steady state (time constant; tau).

$$Y(t)=Y(baseline)+Amp\left[1-e^{-(t-TD)/\tau }\right]$$

where Y(t) represents FBF at any time (t), Y(baseline) is the resting value immediately prior to the onset of exercise, Amp is the amplitude of the response, TD is the time delay, and τ represents the time (i.e., number of duty cycles; tau) required to achieve 63% of the steady state amplitude.

SmO_2_ was fit with a mono-exponential decay in order to assess the time required to reach 37% of steady state (time constant; tau).

$$Y(t)=Y(baseline)-Amp\left[e^{-t/\tau }\right]$$

where Y(t) represents SmO_2_ at any time (t), Y(baseline) is the resting value immediately prior to the onset of exercise, Amp is the amplitude of the response, and τ represents the time (i.e., number of duty cycles; tau) required to achieve 37% of the steady state decay.

### Statistical and time course analysis

Statistical analysis was completed using a combination of JASP (JASP Team, v0.17.2) and SigmaPlot 12.0 (Systat Software, Inc.). Time course analysis was completed using SigmaPlot 12.0 (Systat Software, Inc.). Normality of data was assessed with a Shapiro-Wilk test. Mixed model, two-way RMANOVAs were performed. Following significant F-statistics, Bonferroni corrected *post hoc* t-tests were completed. This analysis approach tested for main effects of arm dominance (non-dominant vs. dominant arm), time (exercise vs. recovery), and biological sex (male vs. female). It also tested for the following interactions: arm dominance and time, arm dominance and sex, time and sex, as well as arm dominance and time and sex. Assumptions of normality and homogeneity of variance in the RMANOVAs were met. For participant characteristics and time course analyses, the dominant arm was compared to the non-dominant arm using paired t-tests or Wilcoxon signed-rank tests as needed while males were compared to females using independent t-tests or Mann-Whitney U tests as needed. Equivalence testing was completed with a frequentist framework. The two one-sided t-test (TOST) method was applied with a 90% confidence interval against the null hypothesis that there is a difference between dominant and non-dominant arms greater than half of a standard deviation. Parametric data are presented as mean ± SD. Non-parametric data are presented as median, interquartile range (Q1-Q3). Statistical significance was set at p<0.05 for all analyses and data are Δ from resting baseline to remove potential variation between people in resting perfusion and highlight the hemodynamic effects of forearm exercise.

## Results

### Participant characteristics and force production

All relevant data are within the paper and its Supporting Information files. Participant characteristics are presented in Table 1. Males were taller (p<0.001) and heavier (p = 0.004) compared to females. A greater proportion of females were right hand dominant compared to males (p = 0.025). Forearm measures and force production data are presented in Table 2. For both the non-dominant and dominant arms, males had a larger forearm girth (both p = 0.002), less skinfold thickness above the *flexor digitorum superficialis* (both p≤0.009) and a greater MVC (both p≤0.002) compared to females. The non-dominant and dominant arms of males did not differ with respect to forearm girth, skinfold thickness, or their MVC (all p>0.1). The non-dominant and dominant arms of females did not differ with respect to forearm girth (p = 0.263); however, their non-dominant arm had a greater skinfold thickness (p = 0.014) as well as a lower MVC (p = 0.003) compared to their dominant arm. As a group, resting brachial artery diameter was smaller in the non-dominant arm (0.33±0.06 vs. 0.34±0.05 cm, p = 0.008). This difference was attributed to a smaller diameter in females (p = 0.016) as males did not differ (p = 0.239).

**Table 1:** Participant characteristics.

Variable	All (n = 20)	Male (n = 10)	Female (n = 10)	P-value
Age (yrs)	23 ± 4	22 (19–27)	22 (20–24)	0.939
Height (cm)	172 ± 11	182 ± 4	163 ± 5*	**<0.001**
Weight (kg)	68 ± 13	74 (72–79)	63 (54–67)*	**0.004**
BMI (kg/m^2^)	22.8 ± 3.1	23.0 ± 3.2	22.6 ± 3.1	0.755
Right-handed (%)	80	60	100	**0.025**
7-day PAR score (METs/wk)	45 ± 22	46 ± 24	44 ± 21	0.880

Parametric variables are presented as mean ± SD. Non-parametric variables are presented as median (Q1-Q3). BMI; body mass index, PAR; physical activity recall. Males were compared to females using an independent t-test or Mann-Whitney U test as needed.

* denotes statistically significant difference between male and female (p<0.05).

**Table 2:** Forearm measures, force production and resting absolute intensity hemodynamics.

	All n = 20; 80% RH			Males (M) n = 10; 60% RH			Females (F) n = 10; 100% RH			M vs. F P-values	
	ND Arm	D Arm	P-value	ND Arm	D Arm	P-value	ND Arm	D Arm	P-value	ND Arm	D Arm
Forearm Girth (cm)	25.1 ± 2.8	25.3 ± 2.8	0.111	26.8 ± 2.5	27.1 ± 2.5	0.287	23.4 ± 1.9	23.6 ± 1.7	0.263	**0.002**	**0.002**
Skinfold Thickness (mm)	2 (2–3)	2 (2–2)	0.167	2 (2–2)	2 (2–2)	0.251	3 (2–4)	2 (2–4)*	**0.014**	**0.001**	**0.009**
MVC (N)	315 ± 140	354 ± 127*	**< 0.001**	409 ± 130	432 ± 123	0.101	220 ± 68	274 ± 69*	**0.003**	**< 0.001**	**0.002**
30%MVC target (N)	94 ± 42	106 ± 38*	**< 0.001**	123 ± 39	129 ± 37	0.101	66 ± 21	82 ± 21*	**0.003**	**< 0.001**	**0.002**
Absolute Force (N)	97 ± 11	98 ± 13	0.606	99 ± 12	96 ± 12	0.415	96 ± 13	101 ± 13*	**0.038**	0.582	0.318
Relative Force (N)	96 ± 44	105 ± 39*	**0.040**	123 ± 42	126 ± 39	0.680	69 ± 24	82 ± 25*	**0.001**	**0.002**	**0.007**
Brachial artery diameter (cm)	0.33 ± 0.06	0.34 ± 0.05	**0.008**	0.37 ± 0.04	0.38 ± 0.05	0.239	0.29 ± 0.04	0.31 ± 0.03	**0.016**	**<0.001**	**<0.001**
FBF (ml/min)	23 (20–43)	28 (23–53)	0.111	34 (22–50)	44 (29–69)	0.168	21 (18–25)	26 (19–28)	0.275	0.075	**0.043**
MAP (mmHg)	89 (87–94)	93 (87–95)	0.739	94 (90–95)	94 (87–95)	0.519	87 (85–88)	92 (88–95)	0.130	**0.009**	0.684
FVC (ml/min/100mmHg)	26 (22–46)	30 (23–58)	0.312	35 (24–48)	48 (30–82)	0.139	24 (20–29)	27 (21–33)	0.846	0.123	0.052
SpO_2_ (%)	99 (99–99)	99 (98–99)	0.120	99 (99–99)	99 (98–99)	0.346	99 (99–99)	99 (98–99)	0.345	0.583	0.524
SmO_2_ (%)	75 ± 8	77 ± 7	0.307	74 ± 8	77 ± 7	0.341	76 ± 9	77 ± 8	0.776	0.575	0.978

Parametric variables are presented as mean ± SD. Non-parametric variables are presented as median (Q1-Q3). MVC; maximal voluntary contraction, RH; right-handed, FBF; forearm blood flow, MAP; mean arterial pressure, FVC; forearm vascular conductance, SpO_2_; oxygen pulse saturation, SmO_2_; skeletal muscle oxygen saturation. Dominant (D) arms were compared to non-dominant (ND) arms using a paired t-test or Wilcoxon signed-rank test as needed. Males were compared to females using an independent t-test or Mann-Whitney U test as needed.

* denotes statistically significant difference between ND and D arm (p<0.05).

As a group, the non-dominant arm had a lower target for relative intensity exercise compared to the dominant arm (94±42 vs. 106±38 N, p<0.001). When partitioned by sex, the non-dominant and dominant arms of males did not differ with respect to their relative intensity targets (123±39 vs. 129±37 N, p = 0.101) nor their relative (123±42 vs. 127±39 N, p = 0.680) or absolute (99±12 vs. 96±12 N, p = 0.415) intensity force production during exercise. In females, the non-dominant arm had a lower target compared to the dominant arm (66±21 vs. 82±21 N, p = 0.003) and a lower relative (69±24 vs. 82±25 N, p = 0.001) and absolute (96±13 vs. 101±13 N, p = 0.038) force production during exercise. Given that relative intensities did not differ between forearms within males, for simplicity, only the absolute results are presented for males. Resting absolute intensity hemodynamics did not differ between arms (Table 2).

### Hemodynamics during absolute intensity forearm exercise

**Fig 2:**
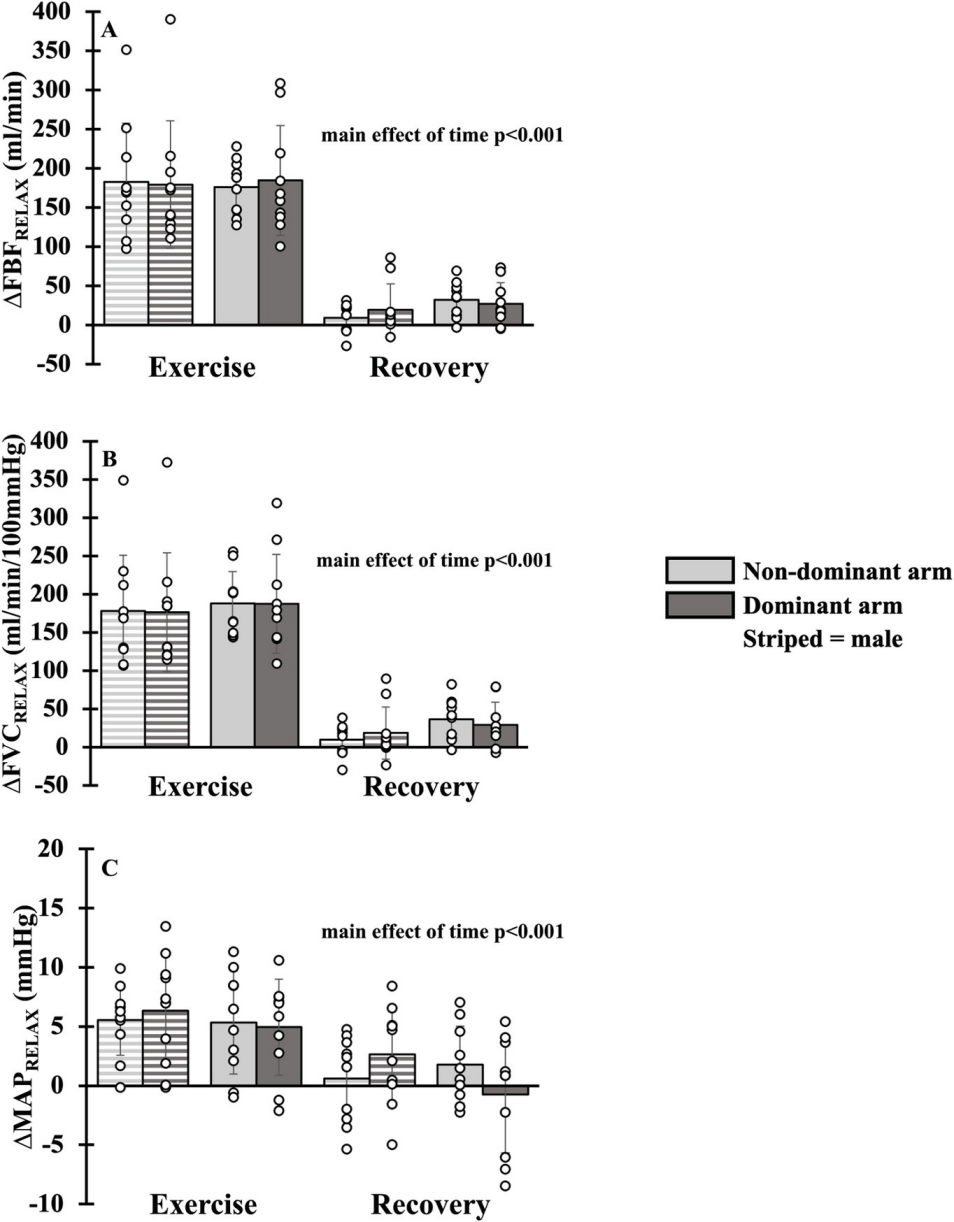
Hemodynamics during absolute intensity forearm exercise. Forearm blood flow (Panel A), forearm vascular conductance (Panel B) and mean arterial blood pressure (Panel C) during exercise and recovery in dominant (dark gray bar) and non-dominant (light gray bar) arms. Data are Δ from rest. Individual responses are shown in open circles. Males are shown with striped bars.

**Fig 3:**
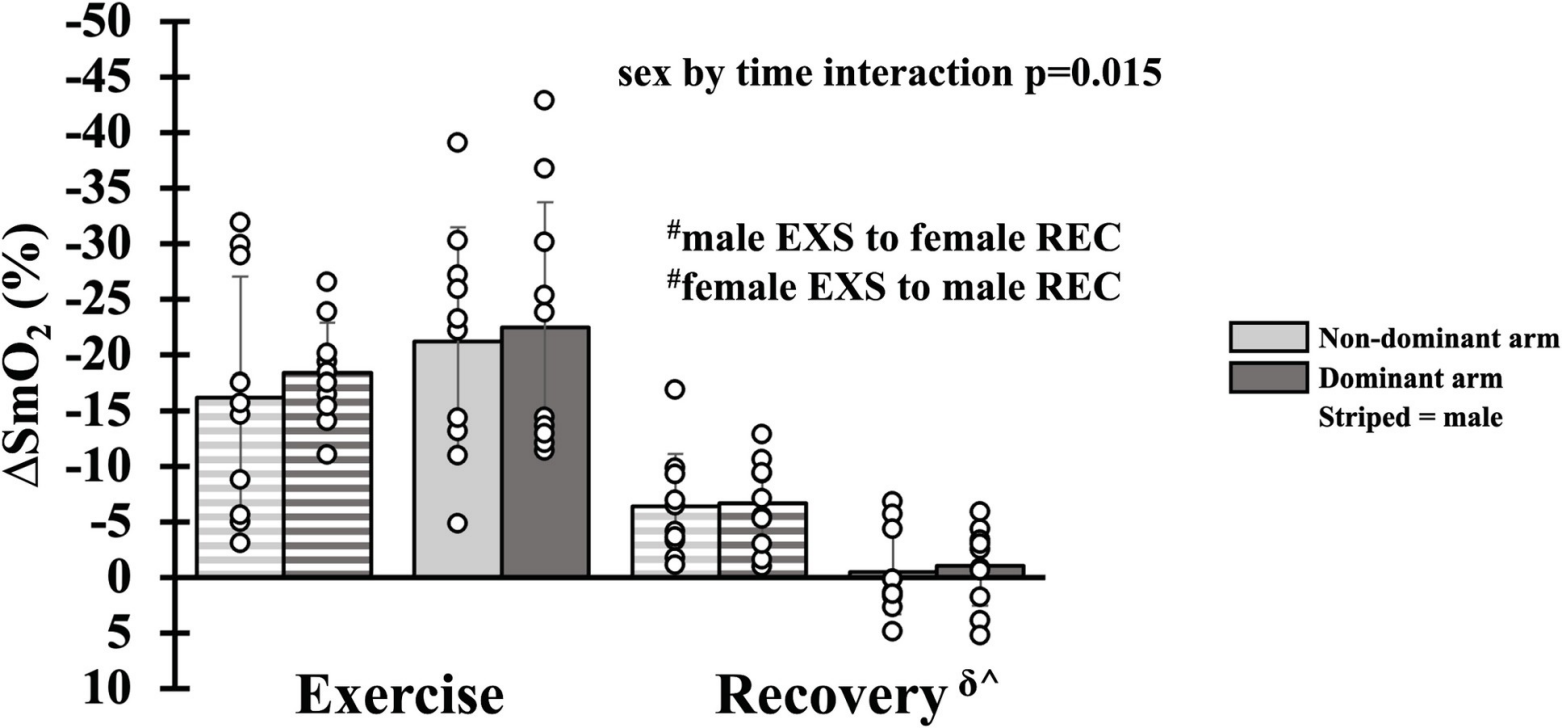
Skeletal muscle oxygen saturation during absolute intensity forearm exercise. Skeletal muscle oxygen saturation during exercise (EXS) and recovery (REC) in dominant (dark gray bar) and non-dominant (light gray bar) arms. Data are Δ from rest. Individual responses are shown in open circles. Males are shown with striped bars. Statistically significant difference (p<0.05) between exercise and recovery: ^δ^For males, ^^^for females, ^#^between males and females.

There was a main effect of time for FBF_RELAX_, FVC_RELAX_ and MAP_RELAX_ with all parameters falling from exercise to recovery (all p<0.001, Fig 2). A main effect of arm dominance was not present for FBF_RELAX_ (p = 0.835), FVC_RELAX_ (p = 0.914) or MAP_RELAX_ (p = 0.986). Additionally, there was no main effect of sex for FBF_RELAX_ (p = 0.611), FVC_RELAX_ (p = 0.309) and MAP_RELAX_ (p = 0.434). A main effect of arm dominance was not present for SmO_2_ (p = 0.506), but there was an interaction of sex and time (p = 0.015) with SmO_2_ rising from exercise to recovery in both males and females (all p<0.005). SmO_2_ during exercise (p = 0.513) and recovery (p = 0.191) were not different between sex (Fig 3). Moreover, FBF_REALX_, FVC_RELAX_ and MAP_RELAX_ were equivalent during exercise (all p_upper_<0.027, p_Lower_<0.021) and recovery (all p_upper_<0.034, p_Lower_<0.027) between the non-dominant and dominant arms. SmO_2_ was equivalent during recovery (p_upper_ = 0.009, p_Lower_ = 0.036) but not during exercise (p_upper_ = 0.005, p_Lower_ = 0.065). For simplicity, mean FBF, FVC and MAP have not been duplicated as the statistical and physiological interpretation was unchanged.

### Hemodynamics during relative intensity forearm exercise in females

**Fig 4:**
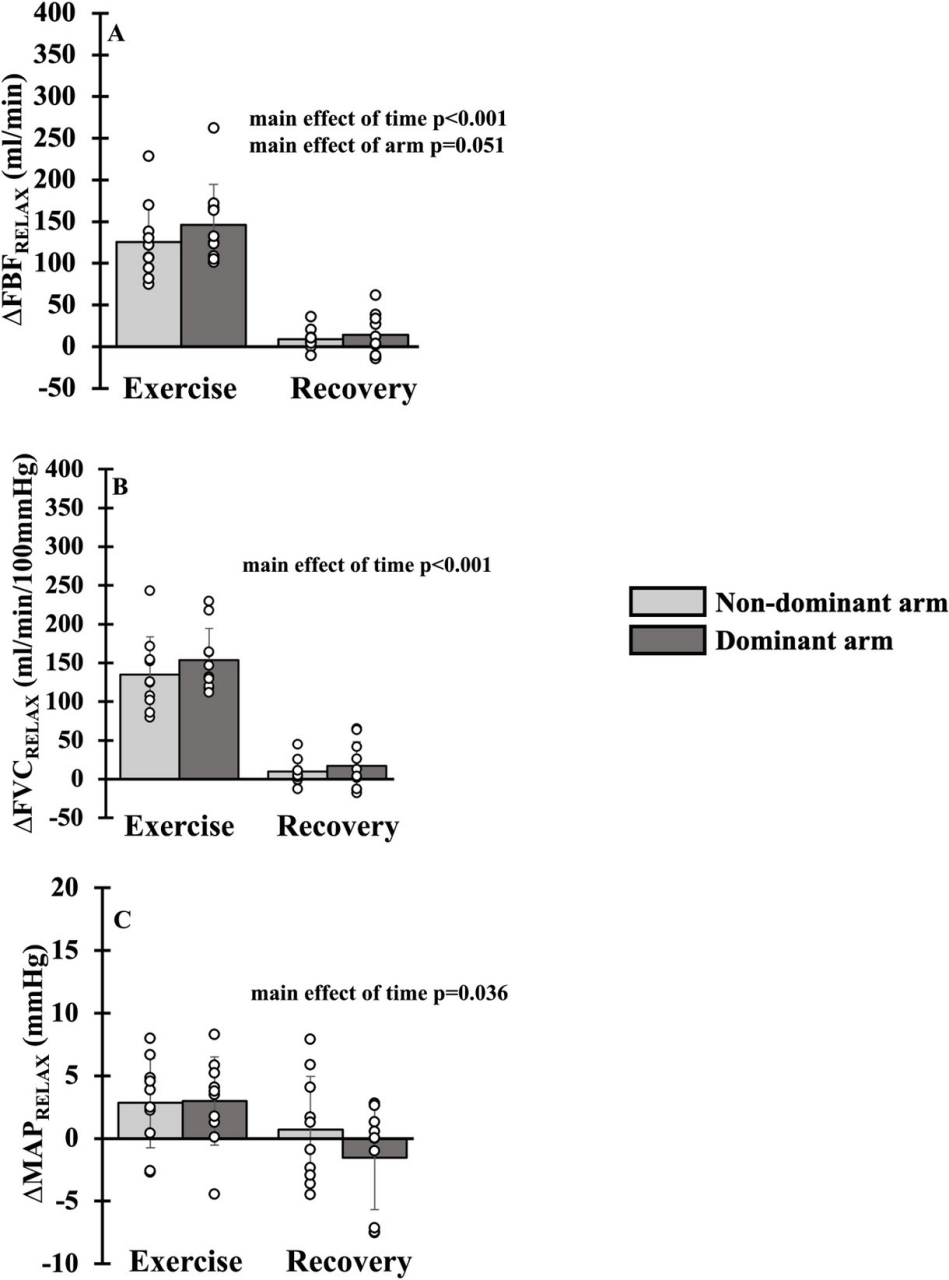
Hemodynamics during relative intensity forearm exercise in females. Forearm blood flow (Panel A), forearm vascular conductance (Panel B) and mean arterial blood pressure (Panel C) during exercise and recovery in dominant (dark gray bar) and non-dominant (light gray bar) arms. Data are Δ from rest. Individual responses are shown in open circles.

**Fig 5:**
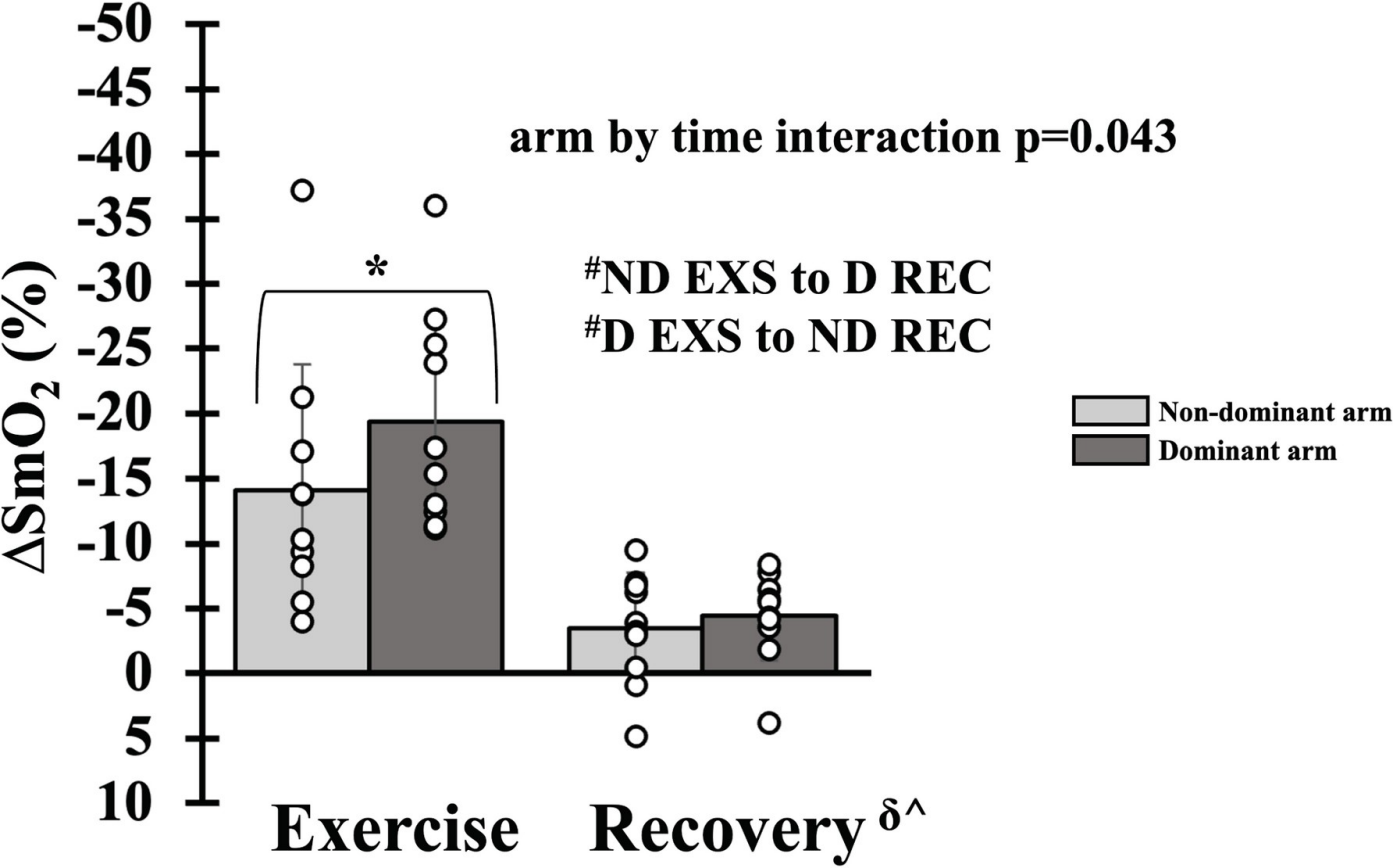
Skeletal muscle oxygen saturation during relative intensity forearm exercise in females. Skeletal muscle oxygen saturation during exercise (EXS) and recovery (REC) in dominant (dark gray bar) and non-dominant (light gray bar) arms. Data are Δ from rest. Individual responses are shown in open circles. Statistically significant difference between exercise and recovery: ^δ^For the non-dominant arm (ND), ^^^for the dominant arm (D). Statistically significant difference between the non-dominant and dominant arm: ^*^During exercise, ^#^between exercise and recovery. p<0.05.

There was a main effect of time for FBF_RELAX_ (p<0.001), FVC_RELAX_ (p<0.001) and MAP_RELAX_ (p = 0.036) with all parameters falling from exercise to recovery (Fig 4). A main effect of arm dominance was statistically absent for FBF_RELAX_ (p = 0.051), while FVC_RELAX_ (p = 0.123) and MAP_RELAX_ (p = 0.281) were not different. There was an interaction of arm dominance and time (p = 0.043, Fig 5) for SmO_2_. During exercise, the non-dominant arm experienced an attenuated reduction in SmO_2_ compared to the dominant arm (-14±10 vs. -19±8%, p = 0.026); however, during recovery, the non-dominant and dominant arms did not differ (-3±4 vs. -4±3%, p = 1). The nadir of SmO_2_ attenuated in the non-dominant arm (-14 (-18 to -11) vs. -20 (-25 to -14) %, p = 0.002). In addition, FBF_REALX_ (p_upper_ = 0.836, p_Lower_ = 0.001), FVC_RELAX_ (p_upper_ = 0.578, p_Lower_ = 0.004), MAP_RELAX_ (p_upper_ = 0.090, p_Lower_ = 0.061) and SmO_2_ (p_upper_<0.001, p_Lower_ = 0.965) were not equivalent during exercise and recovery between then non-dominant and dominant arms. For simplicity, mean FBF, FVC and MAP have not been duplicated as the statistical and physiological interpretation was unchanged.

### Time course analyses

At an absolute intensity, the tau for FBF (p = 0.190) was not different between non-dominant and dominant arms. This observation persisted within both males (p = 0.352) and females (p = 0.432) and when comparing between sex (both p>0.270). Similarly, the tau for SmO_2_ (p = 0.508) was not different between non-dominant and dominant arms. This observation persisted within both males (p = 0.933) and females (p = 0.492) and when comparing between sex (both p>0.696). At a relative intensity in females, the tau for FBF (p = 0.160) and SmO_2_ (p = 0.695) was not different between arms (Table 3). These observations persisted in the context of equivalence testing.

**Table 3:** Time course analyses during exercise.

	All FBF: n = 17; 88% RH SmO_2_: n = 18; 83% RH			Males (M) FBF: n = 7; 71% RH SmO_2_: n = 8; 63% RH			Females (F) FBF: n = 10; 100% RH SmO_2_: n = 10; 100% RH			M vs. F P-values	
	ND Arm	D Arm	P-value	ND Arm	D Arm	P-value	ND Arm	D Arm	P-value	ND Arm	D Arm
Absolute Intensity											
Tau for ΔFBF	23 (16–34)	16 (10–24)	0.190	20 (8–28)	15 (12–17)	0.375	28 (18–33)	21 (9–29)	0.432	0.270	0.475
Tau for ΔSmO_2_	28 (18–63)	22 (17–45)	0.508	27 (20–48)	25 (16–50)	0.933	29 (18–72)	22 (17–43)	0.492	0.696	0.965
Relative Intensity											
Tau for ΔFBF	‐‐‐‐	‐‐‐‐	‐‐‐‐	‐‐‐‐	‐‐‐‐	‐‐‐‐	18 (15–30)	15 (12–21)	0.160	‐‐‐‐	‐‐‐‐
Tau for ΔSmO_2_	‐‐‐‐	‐‐‐‐	‐‐‐‐	‐‐‐‐	‐‐‐‐	‐‐‐‐	16 (13–28)	28 (18–50)	0.695	‐‐‐‐	‐‐‐‐

Parametric variables are presented as mean ± SD. Non-parametric variables are presented as median (Q1-Q3). Tau for ΔFBF (exponential growth): the time in seconds required to reach 63% of steady state. Tau for ΔSmO_2_ (exponential decay): The time in seconds required to reach 37% of steady state. RH; right-handed, FBF; forearm blood flow, SmO_2_; skeletal muscle oxygen saturation. FBF was fit with a mono-exponential model. SmO_2_ was fit with a mono-exponential decay. Dominant (D) arms were compared to non-dominant (ND) arms using a Wilcoxon signed-rank test. Males were compared to females using a Mann-Whitney U test.

* denotes statistically significant difference between ND and D arm (p<0.05).

## Discussion

The human forearm model is commonly employed in physiological investigations exploring vascular function and oxygen delivery. Studies that incorporate this model often use the left or right forearm exclusively for experimental simplicity; however, no previous work examined the effect of arm dominance on non-ischemic exercising forearm hemodynamics in untrained individuals. The primary findings from the present investigation revealed that: 1) At an absolute exercise intensity, the non-dominant and dominant arm did not differ with respect to FBF_RELAX_, FVC_RELAX_, SmO_2_, and the tau for FBF and SmO_2_, and 2) At a relative intensity in females, FBF_RELAX_ was greater and SmO_2_ was reduced in the dominant arm while the tau for FBF and SmO_2_ were not different. The results from this study provide important foundational information about the effect, or lack thereof in some respects, of arm dominance in studies utilizing a forearm model to advance the understanding of cardiovascular control.

### Hemodynamics and skeletal muscle oxygen saturation during exercise

In order to the meet the oxygen demand of metabolically active tissues, a combination of convective (i.e., muscle blood flow) and diffusive (i.e., red blood cell desaturation) oxygen delivery perfuse said tissue [1–3]. During small muscle mass exercise, muscle blood flow has been shown to increase linearly up to 93% of peak power while venous oxygen extraction plateaus between 79 and 93% [26]. During acute, mild intensity small muscle mass rhythmic exercise, elevations in blood flow are not accompanied by large changes in muscle sympathetic nerve activity [27,28]. In order to fully explore the relationship between convective and diffusive oxygen delivery between dominant and non-dominant arms, we employed both absolute and relative exercise intensities that are below those in which a plateau in oxygen extraction may be observed. When exercising at the same fixed intensity, the oxygen demand is constant such that any difference in muscle blood flow between arms may be adjusted by an increase in oxygen extraction in order to meet the muscle’s oxygen demands regardless of the rate of muscle blood flow. When exercising at the same relative intensity as a percentage of MVC, the demand in relation to maximal capacity is consistent. In the present study, the MVC for males was not different resulting in the relative intensity force productions being not different (123±42 vs. 126±39 N, p = 0.680). Therefore, only results from absolute intensity forearm exercise in males was presented and discussed.

#### Absolute exercise intensity

In support of our hypothesis, exercising at a fixed oxygen demand between arms revealed no differences in steady state FBF_RELAX_, FVC_RELAX_, MAP_RELAX_ or SmO_2_. This suggests that the hemodynamic response is unaffected by arm dominance such the contribution of convective and diffusive components to oxygen delivery are not different between arms. This observation extends hemodynamic assessments by Rowley et al. [16] in healthy teenage males in which non-forearm trained individuals demonstrated no difference between arms in peak brachial artery blood flow following ischemic handgrip exercise. Further, the results from the present study align with non-exercising evidence highlighting no difference in brachial artery diameter both at rest and following glyceryl trinitrate administration between arms [16], although we did observe a reduced brachial artery diameter in the non-dominant arm of females. In addition, previous work noted that brachial artery flow mediated dilation in response to cuff inflation was not different between arms in a retrospective analysis in males with 34% of the variability in non-dominant limb flow mediated dilation explained by the dominant limb [17]. Visually, there was seemingly greater variability when one limb had a flow mediated dilation response of >8%, reflective of greater endothelial function [17]. That being said, the present results demonstrating that the active hyperemic response is consistent between arms does not align with the reactive hyperemia response observed in similarly aged and healthy, right-handed males [18]. This difference may be attributed to nature of the hemodynamic stimulus with reactive hyperemia arising from local ischemia while active hyperemia arises from muscle activation. Racquet sports players present quite uniquely as unilaterally forearm trained and suggest that sport specific training, as seemingly opposed to the training associated with habitual activities of daily living, do experience disparate hemodynamic outcomes. Kagaya et al. [15] demonstrated that in response to static handgrip exercise in twenty year-old female tennis players, recovery brachial artery diameter and blood flow is greater in the trained forearm. While the completion of static exercise may impede flow consistently between 10–90% of maximal effort [29] and therefore between limbs, females may experience less contraction induced impairments in blood flow compared to males [30]. The present observations provide support that the hemodynamic response to non-ischemic exercise is equivalent. This observation is particularly noteworthy as conflicting reports have suggested that the ability to effectively match oxygen delivery to oxygen demand may differ substantially between people in response to a perturbation (i.e., local ischemia and perfusion pression reductions) [9,19,20]. While not assessed prospectively in previous work, the results from the present study suggest that differences in arm dominance would not influence the ability to overcome hemodynamic perturbations and match oxygen delivery to oxygen demand in untrained individuals.

#### Relative exercise intensity

While absolute exercise intensities provide a consistent oxygen demand between arms, the relative intensity may differ due to differences in handgrip strength. In the present study, MVC was not different between dominant and non-dominant arms in males and when computed, the relative intensity targets were not different. However, in females, the dominant arm had a greater MVC and as such, a greater relative intensity target compared to the non-dominant arm. While hemodynamic responses were not different at an absolute intensity, selection of a constant relative intensity alleviates the potential intensity effects as a function of maximal capacity. Contrary to our hypothesis, when exercising at a relative intensity in females, FVC_RELAX_ (and MAP_RELAX_) were not different between arms. However, inline with our hypothesis, FBF_RELAX_ was seemingly greater (p = 0.051) in the dominant arm compared to the non-dominant arm. This greater local perfusion is related to the greater force production (82 ± 25 vs. 69 ± 24 N, p = 0.001) and local oxygen demand. When scaled to the relative intensity target, the increase in FBF_RELAX_ at steady state was 1.84 ml/min/N in the dominant and 1.89 ml/min/N in the non-dominant arm (p = 0.460). Despite this similar increase in scaled FBF_RELAX_, contrary to our hypothesis, the non-dominant arm experienced both an attenuated reduction in SmO_2_ that was alleviated during recovery as well as an attenuated nadir (lowest 3 second contraction-relaxation duty cycle) during exercise. Evidence suggests that the capillary beds have a large degree of perfusion heterogeneity [31] alongside up to 50 capillary modules supplying a muscle fibre [32]. As such, the distribution of conduit brachial artery blood flow to the muscle fibre requires the control of 50 arterials [33] and with potential motor unit recruitment and firing behaviour modulated by hand dominance [34] –capillary perfusion and by extension local SmO_2_, may be impacted by arm dominance at the prescribed relative exercise intensities. Future work would be required to confirm forearm muscle activation in this context.

### Time course of forearm hemodynamics during exercise

While steady state measures provide an indication into the eventual matching of oxygen delivery to oxygen demand, the transition enroute to steady state may be important for the extent of an oxygen deficit experienced and subsequent exercise fatigue progression. Aligning with our hypothesis, the time constant tau for FBF and SmO_2_ were not different between arms at both absolute and relative intensities. This observation supports previous work identifying that the mean response time for skeletal muscle blood flow during low and high intensity single leg knee extension exercise [35] as well as FVC during forearm exercise [5] are intensity independent. Previous work has identified that the tau for deoxy-hemoglobin at the *vastus lateralis/medialis* and the rate of pulmonary oxygen consumption during moderate intensity knee extension exercise are similar [36] and reflect a slower alteration of microvascular blood flow relative to oxygen utilization. The present observation identified no difference in tau values between FBF and SmO_2_, which suggests that macro and micro-vascular perfusion are similar in the forearm, at the presently prescribed relatively low intensities, in both arms. As such, habitual forearm training tied to activities of daily living does not influence the rest to exercise transition and resulting oxygen deficit, though future work is required to confirm the relationship between macro, micro-vascular and oxygen uptake between limbs and higher exercise intensities.

### Experimental considerations

While the present study assessed exercise hyperemia and vasodilation, oxygen delivery as the product of muscle blood flow and arterial oxygen content [37,38], was not computed. That being said, arterial oxygen content remains relatively constant throughout exercise [4,39] and as such, elevations in muscle blood flow facilitate increases in oxygen delivery. Given the experimental design in which each participant completed exercise with both dominant and non-dominant arms, arterial oxygen content would be the same in each condition such that any differences in blood flow are interpreted as differences in oxygen delivery. Arm dominance was self-identified, and it is appreciated that the extent of dominance/preference [40] in its entirety for certain activities (e.g., using a hammer or carrying a suitcase) was not assessed in the present study when contextualizing the effect of arm dominance on forearm hemodynamics. That being said, it is unlikely that such an effect would persist across twenty participants. Muscle fibre typing was not completed on the *flexor digitorum superficialis* and while all participants were recreationally active without forearm specific training, potential differences in fibre type composition cannot be entirely eliminated–though evidence in older men suggest the flexor digitorum superficialis is ~50% type I fibres [41]. Females in the present investigation were studied in the follicular (50%) and luteal (40%) phases (10% intrauterine device). Given evidence suggesting that menstrual cycle phase does not influence endothelial function [42,43], with the small effect on macrovascular function attributed to methodological differences in assessments [44], we did not feel it necessary to control for menstrual phase and do not believe menstrual cycle differences between females impact the interpretation of results.

## Conclusion

The human forearm model is commonly employed in physiological investigations exploring local vascular function and oxygen delivery. Although forearm-trained racquet sports athletes exhibit enhanced hemodynamics in their dominant arm, the present investigation has demonstrated that arm dominance in untrained individuals does not impact forearm hemodynamics or SmO_2_ during non-ischemic handgrip exercise. These findings suggest that investigations utilizing a forearm model to advance the understanding of cardiovascular control in untrained individuals may use arms interchangeably within a study design without compromising the physiological interpretation.

## Supporting information

S1 Appendix Manuscript data. (XLSX)

## References

[1] Mendelson AA, Milkovich S, Hunter T, Vijay R, Choi Y-H, Milkovich S, et al. The capillary fascicle in skeletal muscle: Structural and functional physiology of RBC distribution in capillary networks. J Physiol. 2021;599: 2149–68. doi: 10.1113/JP281172 33595111

[2] Tucker WJ, Rosenberry R, Trojacek D, Chamseddine HH, Arena-Marshall CA, Zhu Y, et al. Studies into the determinants of skeletal muscle oxygen consumption: novel insight from near-infrared diffuse correlation spectroscopy. J Physiol. 2019;597: 2887–901. doi: 10.1113/JP277580 30982990 PMC8024923

[3] Boushel R, Ara I, Gnaiger E, Helge JW, González-Alonso J, Munck-Andersen T, et al. Low-intensity training increases peak arm VO2 by enhancing both convective and diffusive O2 delivery. Acta Physiologica. 2014;211: 122–34. doi: 10.1111/apha.12258 24528535

[4] Andersen P, Saltin B. Maximal perfusion of skeletal muscle in man. J Physiol. 1985;366: 233–49. doi: 10.1113/jphysiol.1985.sp015794 4057091 PMC1193029

[5] Saunders NR, Pyke KE, Tschakovsky ME. Dynamic response characteristics of local muscle blood flow regulatory mechanisms in human forearm exercise. J Appl Physiol. 2005;98: 1286–96. doi: 10.1152/japplphysiol.01118.2004 15579568

[6] Hughson RL, Shoemaker JK, Tschakovsky ME, Kowalchuk JM. Dependence of muscle VO2 on blood flow dynamics at onset of forearm exercise. J Appl Physiol. 1996;81: 1619–26. doi: 10.1152/jappl.1996.81.4.1619 8904578

[7] Dinenno FA, Joyner MJ. Combined NO and PG inhibition augments-adrenergic vasoconstriction in contracting human skeletal muscle. Am J Physiol Heart Circ Physiol. 2004;287: 2576–84. doi: 10.1152/ajpheart.00621.2004 15271659

[8] Lind AR, McNicol GW. Local and central circulatory responses to sustained contractions and the effect of free or restricted arterial inflow on post‐exercise hyperaemia. J Physiol. 1967;192: 575–593. doi: 10.1113/jphysiol.1967.sp008318 6058994 PMC1365529

[9] Bentley RF, Walsh JJ, Drouin PJ, Velickovic A, Kitner SJ, Fenuta AM, et al. Absence of compensatory vasodilation with perfusion pressure challenge in exercise: evidence for and implications of the noncompensator phenotype. J Appl Physiol. 2018;124: 374–87. doi: 10.1152/japplphysiol.00952.2016 28706000 PMC5867373

[10] Kirby BS, Carlson RE, Markwald RR, Voyles WF, Dinenno FA. Mechanical influences on skeletal muscle vascular tone in humans: Insight into contraction-induced rapid vasodilatation. J Physiol. 2007;583: 861–74. doi: 10.1113/jphysiol.2007.131250 17495044 PMC2277182

[11] Tschakovsky ME, Rogers AM, Pyke KE, Saunders NR, Glenn N, Lee SJ, et al. Immediate exercise hyperemia in humans is contraction intensity dependent: evidence for rapid vasodilation. J Appl Physiol. 2004;96: 639–44. doi: 10.1152/japplphysiol.00769.2003 14578368

[12] Zaccagni L, Toselli S, Bramanti B, Gualdi-Russo E, Mongillo J, Rinaldo N. Handgrip strength in young adults: Association with anthropometric variables and laterality. Int J Environ Res Public Health. 2020;17: 4273. doi: 10.3390/ijerph17124273 32549283 PMC7345833

[13] Pang J, Tu F, Han Y, Zhang E, Zhang Y, Zhang T. Age-related change in muscle strength, muscle mass, and fat mass between the dominant and non-dominant upper limbs. Front Public Health. 2023;11: 1284959. doi: 10.3389/fpubh.2023.1284959 38074765 PMC10701377

[14] Mori S, Kosaki K, Tagata R, Kon K, Yasuda R, Nishitani N, et al. Acute influences of tennis services on cardiac output and brachial hemodynamics in young male tennis players. J Sci Med Sport. 2022;25: 973–978. doi: 10.1016/j.jsams.2022.10.009 36357270

[15] Kagaya A, Ohmori F, Okuyama S, Muraoka Y, Sato K. Blood flow and arterial vessel diameter change during graded handgrip exercise in dominant and non-dominant forearms of tennis players. Adv Exp Med Biol. 2010;662: 365–70. doi: 10.1007/978-1-4419-1241-1_53 20204817

[16] Rowley NJ, Dawson EA, Birk GK, Cable NT, George K, Whyte G, et al. Exercise and arterial adaptation in humans: Uncoupling localized and systemic effects. J Appl Physiol. 2011;110: 1190–5. doi: 10.1152/japplphysiol.01371.2010 21350023

[17] Thijssen DHJ, Rowley N, Padilla J, Simmons GH, Harold Laughlin M, Whyte G, et al. Relationship between upper and lower limb conduit artery vasodilator function in humans. J Appl Physiol. 2011;111: 244–50. doi: 10.1152/japplphysiol.00290.2011 21512151 PMC3137536

[18] Kadoguchi T, Horiuchi M, Kinugawa S, Okita K. Heterogeneity in the vasodilatory function of individual extremities. Vascular. 2020;28: 87–95. doi: 10.1177/1708538119868411 31402786

[19] Bentley RF, Kellawan JM, Moynes JS, Poitras VJ, Walsh JJ, Tschakovsky ME. Individual susceptibility to hypoperfusion and reductions in exercise performance when perfusion pressure is reduced: evidence for vasodilator phenotypes. J Appl Physiol. 2014;117: 392–405. doi: 10.1152/japplphysiol.01155.2013 24970851 PMC4137234

[20] Perrey S, Tschakovsky ME, Hughson RL. Muscle chemoreflex elevates muscle blood flow and O2 uptake at exercise onset in nonischemic human forearm. J Appl Physiol. 2001;91: 2010–6. doi: 10.1152/jappl.2001.91.5.2010 11641338

[21] Tschakovsky ME, Saunders NR, Webb KA, O’Donnell DE. Muscle blood-flow dynamics at exercise onset: Do the limbs differ? Med Sci Sports Exerc. 2006;38: 1811–8. doi: 10.1249/01.mss.0000230341.86870.4f 17019304

[22] Canadian Society of Exercise Physiology. Get Active Questionnaire: Canadian Society of Exercise Physiology. 2017. Available from: https://csep.ca/wp-content/uploads/2021/05/GETACTIVEQUESTIONNAIRE_ENG.pdf.

[23] Oldfield RC. The assessment and analysis of handedness: The Edinburgh inventory. Neuropsychologia. 1971;9: 97–113. doi: 10.1016/0028-3932(71)90067-4 5146491

[24] Sarkin J, Campbell J, Gross L, Roby J, Bazzo S, Sallis J, et al. Project GRAD seven-day physical activity recall interviewer’s manual. J Mater Sci Surf Eng. 1997;29: S91–102.

[25] Herr MD, Hogeman CS, Koch DW, Krishnan A, Momen A, Leuenberger UA. A real-time device for converting Doppler ultrasound audio signals into fluid flow velocity. Am J Physiol Heart Circ Physiol. 2010;298: H1626–32. doi: 10.1152/ajpheart.00713.2009 20173048 PMC2867441

[26] González-alonso J, Mortensen SP, Jeppesen TD, Ali L, Barker H, Damsgaard R, et al. Haemodynamic responses to exercise, ATP infusion and thigh compression in humans: Insight into the role of muscle mechanisms on cardiovascular function. J Physiol. 2008;586: 2405–17. doi: 10.1113/jphysiol.2008.152058 18339690 PMC2479567

[27] Victor RG, Seals DR. Reflex stimulation of sympathetic outflow during rhythmic exercise in humans. Am J Physiol Heart Circ Physiol. 1989;257: H2017–24. doi: 10.1152/ajpheart.1989.257.6.H2017 2603985

[28] Wang HJ, Zucker IH, Wang W. Muscle reflex in heart failure: The role of exercise training. Front Physiol. 2012;3: 398. doi: 10.3389/fphys.2012.00398 23060821 PMC3464681

[29] Osada T, Mortensen SP, Rådegran G. Mechanical compression during repeated sustained isometric muscle contractions and hyperemic recovery in healthy young males. J Physiol Anthropol. 2015;34: 36. doi: 10.1186/s40101-015-0075-1 26520798 PMC4628366

[30] Hammer SM, Sears KN, Montgomery TR, Olmos AA, Hill EC, Trevino MA, et al. Sex differences in muscle contraction-induced limb blood flow limitations. Eur J Appl Physiol. 2024;124: 1121–1129. doi: 10.1007/s00421-023-05339-5 37889287

[31] Ellis CG, Wrigley SM, Groom AC. Heterogeneity of red blood cell perfusion in capillary networks supplied by a single arteriole in resting skeletal muscle. Circ Res. 1994;75: 357–68. doi: 10.1161/01.res.75.2.357 8033345

[32] Emerson GG, Segal SS. Alignment of microvascular units along skeletal muscle fibers of hamster retractor. J Appl Physiol. 1997;82: 42–8. doi: 10.1152/jappl.1997.82.1.42 9029196

[33] Murrant CL, Lamb IR, Novielli NM. Capillary endothelial cells as coordinators of skeletal muscle blood flow during active hyperemia. Microcirculation. 2017;24. doi: 10.1111/micc.12348 28036147

[34] Adam A, De Luca CJ, Erim Z. Hand dominance and motor unit firing behavior. J Neurophysiol. 1998;80: 1373–82. doi: 10.1152/jn.1998.80.3.1373 9744946

[35] Jones AM, Krustrup P, Wilkerson DP, Berger NJ, Calbet JA, Bangsbo J. Influence of exercise intensity on skeletal muscle blood flow, O2 extraction and O2 uptake on-kinetics. J Physiol. 2012;590: 4363–76. doi: 10.1113/jphysiol.2012.233064 22711961 PMC3473291

[36] Dumanoir GR, Delorey DS, Kowalchuk JM, Paterson DH. Kinetics of VO2 limb blood flow and regional muscle deoxygenation in young adults during moderate intensity, knee-extension exercise. Eur J Appl Physiol. 2010;108: 607–17. doi: 10.1007/s00421-009-1263-7 19882164

[37] Hogan MC, Sad S, Arthur PG, Sadi Kurdak S. Effect of gradual reduction in 02 delivery on intracellular homeostasis in contracting skeletal muscle. J Appl Physiol. 1996;80: 1313–21. doi: 10.1152/jappl.1996.80.4.1313 8926261

[38] Mortensen SP, Dawson EA, Yoshiga CC, Dalsgaard MK, Damsgaard R, Secher NH, et al. Limitations to systemic and locomotor limb muscle oxygen delivery and uptake during maximal exercise in humans. J Physiol. 2005;566: 273–85. doi: 10.1113/jphysiol.2005.086025 15860533 PMC1464731

[39] Sun X-G, Hansen JE, Stringer WW, Ting H, Wasserman K. Carbon dioxide pressure-concentration relationship in arterial and mixed venous blood during exercise. J Appl Physiol. 1985;90: 1798–810. doi: 10.1152/jappl.2001.90.5.1798 11299270

[40] Elias LJ, Bryden MP, Bulman-Fleming MB. Footedness is a better predictor than is handedness of emotional lateralization. Neuropsychologia. 1998;36: 37–43. doi: 10.1016/s0028-3932(97)00107-3 9533385

[41] Meznaric M, Čarni A. Characterisation of flexor digitorum profundus, flexor digitorum superficialis and extensor digitorum communis by electrophoresis and immunohistochemical analysis of myosin heavy chain isoforms in older men. Annals of Anatomy. 2020;227: 151412. doi: 10.1016/j.aanat.2019.151412 31408678

[42] D’Urzo KA, King TJ, Williams JS, Silvester MD, Pyke KE. The impact of menstrual phase on brachial artery flow-mediated dilatation during handgrip exercise in healthy premenopausal women. Exp Physiol. 2018;103: 291–302. doi: 10.1113/EP086311 29083061

[43] Shenouda N, Priest SE, Rizzuto VI, MacDonald MJ. Brachial artery endothelial function is stable across a menstrual and oral contraceptive pill cycle but lower in premenopausal women than in age-matched men. Am J Physiol Heart Circ Physiol. 2018;315: H366–H374. doi: 10.1152/ajpheart.00102.2018 29727219

[44] Williams JS, Dunford EC, MacDonald MJ. Impact of the menstrual cycle on peripheral vascular function in premenopausal women: Systematic review and meta-analysis. Am J Physiol Heart Circ Physiol. 2020;319: H1327–H1337. doi: 10.1152/ajpheart.00341.2020 33064553

